# Primary hydatid cyst of the small intestine masquerading as intestinal duplication in a child

**DOI:** 10.11604/pamj.2020.36.83.22815

**Published:** 2020-06-12

**Authors:** Sameh Tlili, Youssef Hellal, Rabia Ben Abdallah, Aida Daib, Malek Boughdir, Fatma Trabelsi, Shanaz Abid, Khaoula Ben Hassine, Wafa Koubaa, Youssef Gharbi, Nejib Kaabar

**Affiliations:** 1 Department of Pediatric Surgery, Habib Thameur Hospital, Tunis, Tunisia,; 2 Department of Pathology, Habib Thameur Hospital, Tunis, Tunisia

**Keywords:** Hydatid cyst, *Echinococcus granulosus*, surgery, small intestine, children

## Abstract

Hydatid disease or hydatidosis is a worldwide zoonosis disease caused by the tapeworm of *Echinococcus granulosus* and still widely endemic in Tunisia especially in rural areas where the sheep-dog cycle is dominant. It is an important public health problem in the pediatric age group causing significant morbidity and mortality. We report a case of primary hydatid cyst of the small intestine in a child and we want to highlight the difficulty that we meet in the diagnosis despite the contribution of imaging.

## Introduction

Cystic echinococcosis is a widespread zoonotic parasitic disease especially in Tunisia which is one of the most endemic countries in the Mediterranean areas [1]. In rural areas, the close association of people with sheep and dogs and unavailability of clean potable water supply makes a region endemic to the disease. Primary peritoneal hydatidosis is a rare finding especially in children where hydatid cysts were found primarily located in the lung followed by the liver. Small intestine is an unusual site of hydatid cyst and no report has been published describing this condition in children. Here we present an interesting case of a 7-year-old boy with a primary isolated hydatid cyst of the small intestine masquerading as intestinal duplication.

## Patient and observation

**Figure 1:**
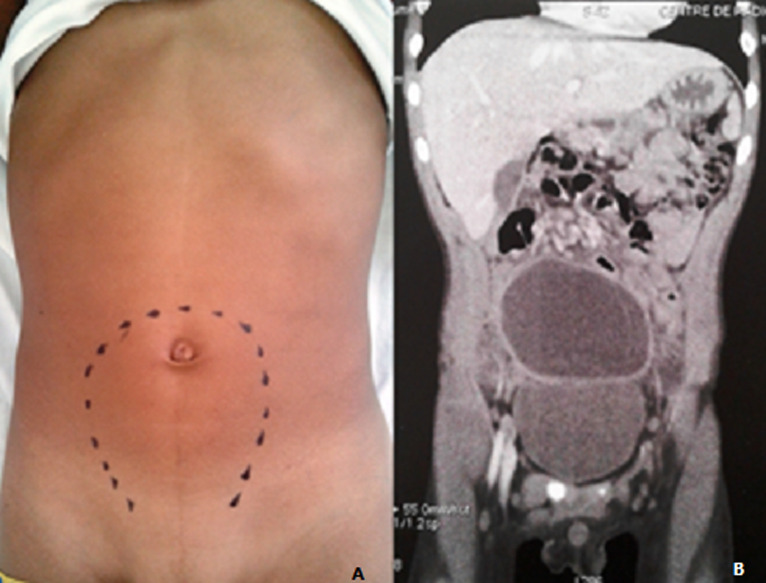
abdominal examination indicating the site of the mass and the contrast enhanced CT image showing a cystic mass above the bladder (A,B)

A 7-year-old boy originated from the governorate of Zaghouan presented to our emergency with lower abdominal pain, vomiting since three days without fever. The patient medical history was unrevealing with no history of trauma. Abdominal examination revealed a tender mobile mass in the hypogastrium reaching up to the umbilicus. The rest of his physical examination was unremarkable. Laboratory investigation showed haemoglobin of 11,5g/dl, total leucocyte count was 10,100/mm^3^ with a differential of 67% neutrophils, 29% lymphocytes, and 3% monocytes. The C-reactive protein concentration and erythrocyte sedimentation rate were 59mg/l and 110mm/h respectively. The hydatid serology (ELISA technique) was negative. A chest X-ray and an abdominal plain radiography showed no pathological appearance. Abdominal ultrasonography (USG) revealed a large septate cystic mass in the right iliac fossa with no free fluid in the peritoneal cavity. Liver and spleen were normal. Contrast enhanced computed tomography of the abdomen detected the cystic mass located in the hypogastria and the right iliac fossa measuring 8,5 X 8 X 4,5cm with water density and having well defined borders. This mass was unilocular with internal septa and shows post contrast enhancement of the cyst wall (Figure 1). Considering the age of the patient and the presence of a large intraabdominal cystic mass, enteric duplication cyst or mesenteric cyst was first considered in differential diagnosis and the patient was operated on.

**Figure 2:**
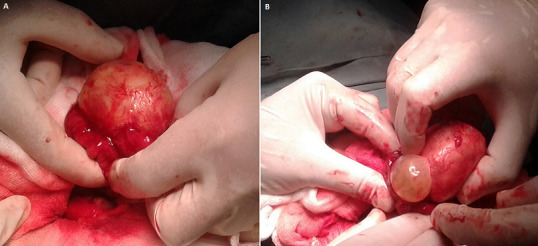
operative picture of the mass surrounded by the small bowel and the appearance of a cyst being extruded (A,B)

**Figure 3:**
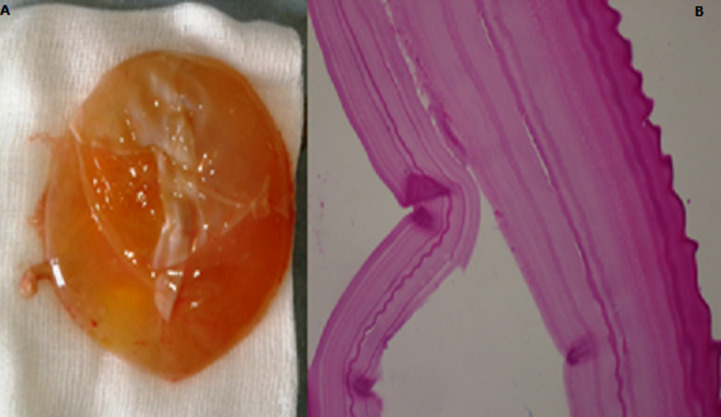
the cyst with presence of the pearly gelatinous cyst wall and histopathologic findings showing cystic structures with laminating fibrous wall (A,B)

During diagnostic laparoscopy a large hypogastric mass filling the lower abdomen obscuring the view with dense adhesions was noticed, however the appendix was located in the sub-hepatic region with no pathologic signs. Conversion was needed due to dense omental adhesions, to severe tissue inflammation around the mass and to intraoperative technical difficulty. On laparotomy, after releasing adhesions, intraoperative findings showed a fibrous whitish mass mainly arising from the small intestine, closely adherent to the adjacent loop (Figure 2). A small incision expelled a cystic mass containing clear fluid which was carefully extruded with its surrounding cheesy material. Segmental intestinal resection was performed with primary anastomosis which was located 70cm from the Bauhin´s valve. Incidental appendicectomy was not accomplished. Abdominal cavity and the whole intestine were then inspected and showed no other abnormality. Histopathology report with hematoxylin and eosine (H&E) staining confirmed the diagnostic and showed features consistent with hydatid cyst with fragments of laminated membrane with no protoscolices (Figure 3). Post operative period was uneventful and the patient was discharged 10 days later with advice to attend regular follow ups.

## Discussion

Hydatid disease caused by *Echinococcus granulosus* remains a public health problem through-out the world despite the deployed prevention programs. Life cycle of *E. granulosus* involves usually dogs as definitive host and sheeps as an intermediate one. Humans are accidental intermediate hosts infected through ingestion of water or food contaminated with dog faeces or through direct contact with animal hair that retain eggs on their coat [2]. Gastric and enteric digestion of eggs releases the embryos which subsequently attach to the duodenal or jejuna, penetrates the intestinal wall (cystic pulmonary hydatidosis) and are then transported by the bloodstream or the lymphatic vessels to various organs. Our patient declared subsequently a direct contact with farm animal and consumption of unwashed vegetal. The most common location of hydatid cysts among children are the lung followed by the liver. Hydatid cyst can occur at any site of the body [3], peritoneal cavity is not spared too. Primary peritoneal hydatid cyst is uncommon without any focuses in other organs. Primary hydatid cysts of gastrointestinal tract involving stomach, small intestine and the colon are exceedingly rare and few cases have been published [4-6]. This may appear surprising since it can be infected in the oral route. Small intestine is an unusual site of primary hydatid cyst.

The mechanism of infestation remains not clear. Some authors suggested that a small primary hydatid cyst of the liver may rupture and then undergo spontaneous resolution, while their content gets seeded into the abdomen [7]. Asymptomatic incubation period may last for many years. Clinical presentation depends on the location, the diameter and the mass effect of enlarging abdominal cyst. Symptoms are often absent and hydatid cyst is detected only incidentally by imaging study, but sometimes it can masquerade other diseases [8]. In our patient, symptoms started to be noticed only when the mass became significantly large in size. Ultrasonography of the abdomen is the most common first-line radiological investigation performed to determine the organ of origin and to characterize the hydatid cyst (dimension, vitality) [9]. Computed tomography (CT) is also a modality of choice in affected patients since it allows imaging of the entire abdomen and pelvis, gives more precise information regarding size, location, adherence to neighbouring structures. However, unilocular type 1 cysts are difficult to differentiate from mesenteric cyst and intestinal duplication cyst [10]. In the present study the exact origin of the cyst was not picked up neither by ultrasonography nor by the CT scan as the mass was large obscuring the anatomy in the hypogastria region. Serology tests have less interest due to its high false positive and false negative rate. Surgical resection is the only curative treatment. Laparatomy is the most common surgical approach.

The goal of the surgery is to remove the cyst without any spillage. It is very important that a correct preoperative diagnosis is made since all precautions must be taken to prevent dissemination. In fact, prophylactic measures such as irrigation with scolicidal solution, protecting the field of surgery by towel soaked with hypertonic saline and albendazole systemic chemotherapy are strongly recommended. In our case through a pfannenstiel incision, the whole cyst was enucleated without any spillage than a segmental resection of the pathologic intestine was performed. Histopathological examination is the final confirmatory diagnosis tool for the identification of the causative agents. A definitive diagnosis of hydatid cyst is confirmed by identification of protoscolices, refractile hooks or fragments of laminated membrane. The cyst can be fertile or sterile like it was in our case. The exact time required for development of protoscoleces within cysts in the human host is not known, but a proportion of cysts does not produce protoscoleces and remain sterile [11]. Hydatid disease at unusual sites carries a diagnostic challenge even in endemic areas. In our report, preoperative clinical examination radiological and serological studies did not give any definite lead toward the diagnosis of hydatid disease. The final diagnosis was done after surgery on the histopathological findings. The current case underlines the possibility of the astonishing resemblance between the clinical and radiological manifestation of the hydatid cyst and intestinal duplication which make the preoperative diagnosis difficult.

## Conclusion

The involvement of small intestine especially as a primary localization of hydatid cyst is a rare finding. Hydatid cyst should always be considered in the differential diagnosis of all intraabdominal cystic mass especially among children who live in endemic areas. This case establishes that hydatid cyst can be found in all parts of the body and this should always be taken into account in the differential diagnosis of cystic lesions.
